# Mouse Model Established by Early Renal Transplantation After Skin Allograft Sensitization Mimics Clinical Antibody-Mediated Rejection

**DOI:** 10.3389/fimmu.2018.01356

**Published:** 2018-07-04

**Authors:** Daqiang Zhao, Tao Liao, Siwen Li, Yannan Zhang, Haofeng Zheng, Jing Zhou, Fei Han, Yu Dong, Qiquan Sun

**Affiliations:** ^1^Division of Kidney Transplantation, Department of Surgery, Third Affiliated Hospital of Sun Yat-sen University, Guangzhou, China; ^2^Department of Pathology, Third Affiliated Hospital of Sun Yat-sen University, Guangzhou, China; ^3^Department of Pathology, First Affiliated Hospital of Sun Yat-sen University, Guangzhou, China

**Keywords:** kidney transplantation, antibody-mediated rejection, donor-specific antibody, animal model, mice

## Abstract

Antibody-mediated rejection (AMR) is the main barrier to renal graft survival, and mouse renal AMR models are important to study this process. Current mouse models are established by priming the recipient to donor skin for over 7 days before kidney transplantation. The robustness of AMR in these cases is too strong to mimic clinical AMR and it is unclear why altering the priming times ranging from 7 to 91 days fails to reduce the AMR potency in these models. In the present study, we found that the donor-recipient combination and skin graft size were determinants of donor-specific antibody (DSA) development patterns after skin transplantation. DSA-IgG was sustained for over 100 days after skin challenge, accounting for an identical AMR robustness upon different skin priming times over 7 days. However, decreasing the skin priming time within 7 days attenuated the robustness of subsequent renal allograft AMR in C3H to Balb/c mice. Four-day skin priming guaranteed that recipients develop acute renal AMR mixed with a high ratio of graft-infiltrating macrophages, renal grafts survived for a mean of 6.4 ± 2.1 days, characterized by typical AMR histological changes, such as glomerulitis, peritubular capillary (PTC) dilation, and capillaritis, deposition of IgG and C3d in PTCs, but less prevalence of microthrombus, whereas the cellular rejection histological change of tubulitis was absent to mild. With this scheme, we also found that the renal AMR model can be developed using common mouse strains such as C57BL/6 and Balb/c, with mean prolonged renal graft survival times of 14.4 ± 5.0 days. Finally, we proved that donor-matched skin challenge after kidney transplantation did not strongly affect DSA development and kidney graft outcome. These findings may facilitate an understanding and establishment of mouse renal allograft AMR models and promote AMR-associated studies.

## Introduction

Antibody-mediated rejection (AMR) has been recognized as the leading cause of kidney graft failure, which accounts for 30–50% of acute rejection episodes in kidney transplantation and over 60% of death-censored late graft loss (1–3). The high incidence and difficulty in treating AMR underscores the need to identify the mechanisms underlying renal allograft AMR in order to develop more efficacious therapeutic strategies to prevent or attenuate antibody-mediated graft injury.

A rodent model of kidney transplantation is useful as an investigational method to understand the mechanism and treatment of rejection (4, 5). Renal allograft AMR models have been established in CCR5^(−/−)^ mice, which spontaneously generate donor-specific antibodies (DSAs) after transplantation (6, 7), and in immunodeficient mice by passive transfer of DSA (8). In wild-type mouse recipients, donor-matched skin graft sensitization prior to kidney transplantation is usually used to induce renal allograft AMR. However, skin graft-induced renal allograft AMR tends to be too severe to facilitate studies on AMR intervention due to early recipient death events, and it is unclear why altering the skin priming times ranging from 7 to 91 days has no impact on the robustness of renal allograft AMR in these models (9–12). The effects of tuning the skin priming time within the early 7-day frame on the potency of subsequent renal graft AMR is still unknown. In this study, we address these questions and establish a less potent renal allograft AMR mouse model, by following the impact of donor-recipient combination, skin graft size, the interval between skin and kidney grafts, and their transplant sequence on DSA generation and the robustness of renal graft AMR.

## Materials and Methods

### Reagents

The primary antibodies used for immunohistochemical staining were purchased from R&D Systems (Minneapolis, MN, USA): anti-C3d; Bethyl Laboratories (TX, USA): anti-IgG; the secondary antibodies were purchased from Beyotime Biotechnology (Shanghai, China): HRP-labeled anti-anti-C3d and HRP-labeled streptavidin. The antibodies used for flow cytometric analysis were purchased from BioLegend (San Diego, CA, USA): BV570-CD45, FITC-CD4, APC-F4/80, BV421-B220; eBioscience (San Diego, CA, USA): eFluor 450-CD8a, Alexa Fluor 700-CD3, PE-CD49b; BD Biosciences: PE-Cy7-CD11b; Abcam (Cambridge, England): anti-IgG; and Jackson ImmunoResearch Laboratories (West Grove, PA, USA): anti-IgM.

### Mice

Male C3H (H-2k), C57BL/6 (H-2b), and BALB/C (H-2d) mice were purchased from Beijing Vital River Laboratory Animal Technology Co., Ltd. and were used at the age of 8–12 weeks. All mice were housed in a specific pathogen free facility at Sun Yat-sen University and all animal experiments were performed in accordance with the Guide for the Care and Use of Laboratory Animals (National Institutes of Health publication no. 80-23, revised 1996) and according to the Sun Yat-sen University Institutional Ethical Guidelines for animal experiments.

### Transplant Surgery

Full-thickness skin grafts with sizes of 8–10 × 10 mm^2^ or 4–5 × 5 mm^2^ from the tails of donor mice were transplanted onto the dorsal area of recipient mice. Murine kidney transplantation models were established as described previously (13). The donor left kidney was harvested for transplantation. For recipient procedures, transplant and bilateral native kidney removal were done simultaneously. Briefly, the right native kidney was removed first after opening the abdomen, and the donor kidney was transplanted and positioned on the right, and finally, the left native kidney was removed before closing the abdomen. Graft rejection leading to death was used as the study endpoint whereas mice with long-term surviving grafts were euthanized at postoperative day 100.

### Experimental Groups

Recipient mice were assigned to the groups showed in Tables 1 and 2.

**Table 1:** Skin transplant groups.

Groups	Graft size (mm^2^)	Donor-recipient combination
1	8–10 × 10	C3H-Balb/c
2	8–10 × 10	C57BL/6-Balb/c
3	8–10 × 10	Balb/c-C3H
4	8–10 × 10	Balb/c-C57BL/6
5	4–5 × 5	C57BL/6-Balb/c

**Table 2:** Kidney transplant groups.

Groups	Descriptions
C3H-Balb/c, nonprimed	Balb/c mice were transplanted with C3H kidneys without skin transplant
C3H-Balb/c, primed-3d	Balb/c mice were transplanted with C3H skins on day 3 prior to receiving C3H kidneys
C3H-Balb/c, primed-4d	Balb/c mice were transplanted with C3H skins on day 4 prior to receiving C3H kidneys
C3H-Balb/c, primed-7d	Balb/c mice were transplanted with C3H skins on day 7 prior to receiving C3H kidneys
C57BL/6-Balb/c, nonprimed	Balb/c mice were transplanted with C57BL/6 kidneys without skin transplant
C57BL/6-Balb/c, primed	Balb/c mice were transplanted with C57BL/6 skins on day 7 prior to receiving C57BL/6 kidneys
C57BL/6-Balb/c, skin Tx	Balb/c mice were transplanted with C57BL/6 skins on day 7 after receiving C57BL/6 kidneys

*All skin grafts were 8–10 × 10 mm^2^ in size*.

### Circulating DSAs Assay

The levels of circulating DSA-IgG and IgM in recipient sera at the indicated time points were assessed by flow cytometry as described previously (10). Briefly, recipient sera were incubated with donor splenocytes at 37°C for 30 min, and the washed cells were then incubated with FITC-labeled goat anti-mouse IgG and rhodamine red-conjugated goat anti-mouse IgM at 4°C for 1 h. Cells were analyzed by flow cytometry with results expressed as mean fluorescence intensity to reflect individual serum DSA levels.

### Histology and Immunohistochemistry

At the time of rejection or on the indicated day after kidney transplantation, the kidney grafts were harvested, formalin-fixed, and paraffin-embedded. Tissue samples were sectioned at 4 µm, deparaffinized and rehydrated, followed by hematoxylin and eosin (H&E) or periodic acid-Schiff (PAS) staining. For immunohistochemical staining, the paraffin-embedded kidney tissues were cut into 4-µm sections, deparaffinized in xylene, rehydrated through graded ethanol, then subjected to quenching of endogenous peroxidase activity in 0.3% hydrogen peroxide, antigen retrieval by microwave heating in 10 mM citrate buffer (pH 6.0), and overnight incubation with primary antibodies against C3d or IgG at 4°C followed by detection using HRP-labeled donkey anti-goat secondary antibody or HRP-labeled streptavidin at 37°C for 30 min. After washing, the tissue sections were stained with diaminobenzidine (DAB) solution and counterstained with hematoxylin. The sections were examined for the severity and category of rejection by light microscopy based on the Banff criteria: 0, absent; 1, mild; 2, moderate; 3, prominent (14).

### Flow Cytometry

Fresh kidney grafts were milled gently in phosphate-buffered saline supplemented with 1% heat-inactivated fetal bovine serum using a needle of a 10-ml syringe before pressing through a 200-mesh nylon screen. The collected cells were then stained with fluorochrome-conjugated antibodies against CD45, CD3, CD4, CD8a, B220, CD49b, CD11b, F4/80, and acquired on a FACSCalibur flow cytometer with Cell Quest Software (BD Biosciences). Data were analyzed with FlowJo Software (Tree Star, Ashland, OR, USA).

### Statistical Analysis

Student’s *t*-test was utilized to compare the differences between two groups. Graft survival among groups was compared using the log-rank test. Data are shown as mean ± SD. Differences with *P*-values <0.05 were considered statistically significant.

## Results

### DSAs Induced by Skin Graft Sensitization Were Affected by Donor-Recipient Combination and Skin Graft Size and Sustained for Long Time

**Figure 1:**
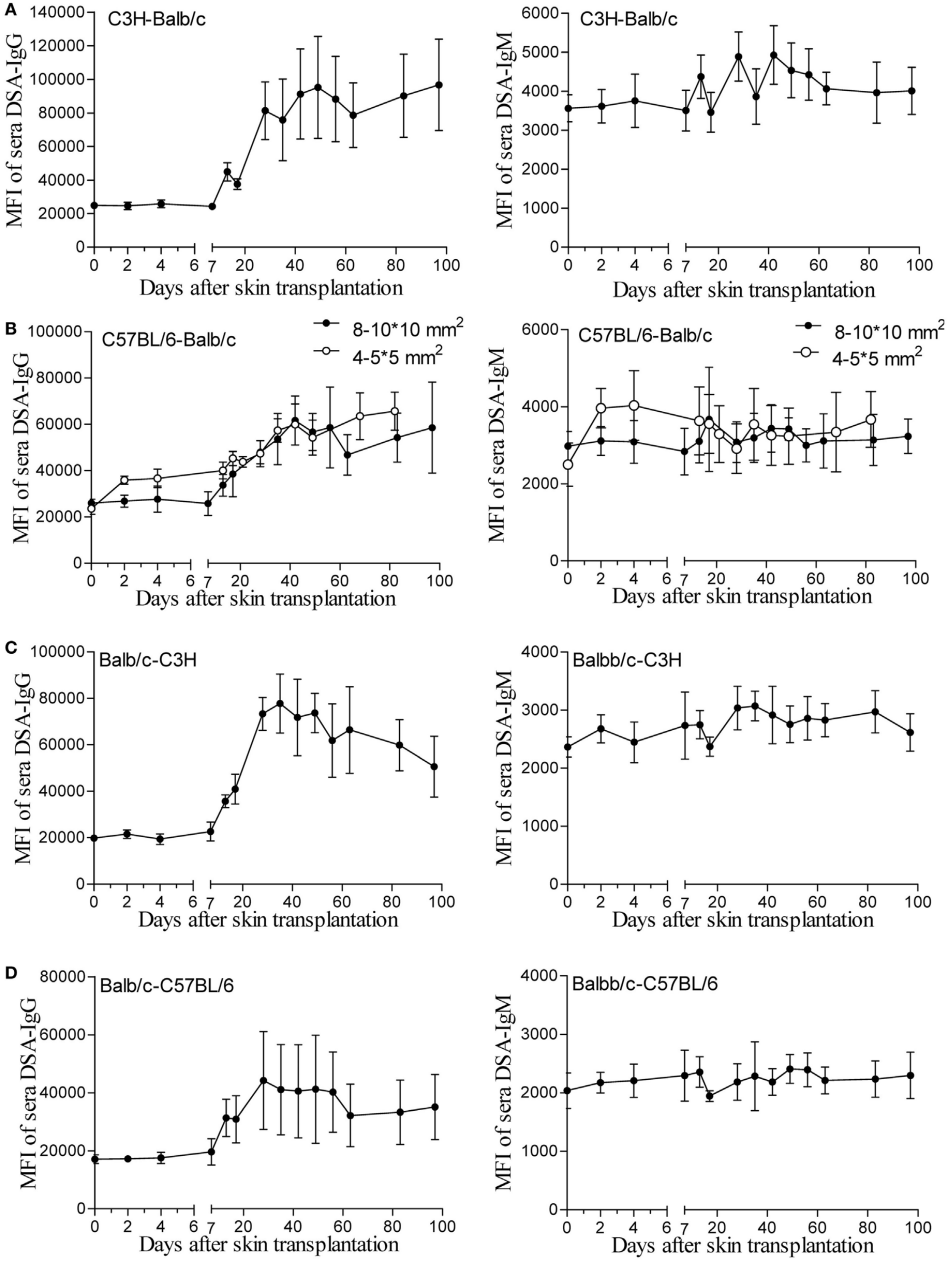
Donor-specific antibodies (DSAs) induced by skin graft sensitization were affected by donor-recipient combination and skin graft size and were sustained for a long time. Skin grafts of size 8–10 × 10 mm^2^ were transplanted onto the back of recipient mice. Recipient sera collected at the indicated time points were reacted with donor spleen cells and evaluated by immunofluorescence and flow cytometry. Levels of detected IgG and IgM antibodies are expressed as mean fluorescence intensity (MFI) ± SD (*n* = 6). **(A)** C3H-Balb/c. **(B)** C57BL/6-Balb/c, skin grafts of size 4–5 × 5 mm^2^ were also applied for comparison with the grafts measuring 8–10 × 10 mm^2^. **(C)** Balb/c-C3H. **(D)** Balb/c-C57BL/6.

One hypothesis for unaltered renal allograft AMR robustness with skin priming time is that the DSA titers after different times of skin priming are sustained for a long time at high enough levels to mediate equally severe rejection after kidney transplantation. To test this, we continuously followed the changes in circulating DSAs by flow cytometry for 100 days after skin transplantation in four different mouse donor-recipient combinations, namely C3H-Balb/c, C57BL/6-Balb/c, Balb/c-C3H, and Balb/c-C57BL/6. As shown in Figure 1, with a graft size of 8–10 × 10 mm^2^, the DSA-IgG levels conformably began to rise on day 7 after transplantation and were increasingly higher from then onward till they reached the peak time in recipient mice from all groups, and were maintained at near peak levels after reaching the highest point within 100 days. The peak times for the DSA-IgG levels in recipients from the above four combinations were posttransplant day 49, 42, 35, and 28, respectively, and the fold increases in the DSA-IgG level at peak times to the baseline levels were 3.82, 2.34, 3.94, and 2.58, respectively. By contrast, the rise in DSA-IgM was extremely slight and exhibited fluctuation within 100 days after skin transplantation in the four different combinations. Compared to the graft size of 8–10 × 10 mm^2^, the 4–5 × 5 mm^2^ skin graft displayed greater efficiency at sensitizing recipients to generate circulating DSAs, which manifested as both DSA-IgG and IgM levels that started to rise much earlier, at day 2 after transplantation; however, from postoperative day 7 onward, this discrepancy resulting from a different graft size was gradually leveled out (Figure 1B).

### Modulating the Robustness of Renal Allograft AMR by Tuning the Priming Time at an Early Stage After Skin Sensitization

**Figure 2:**
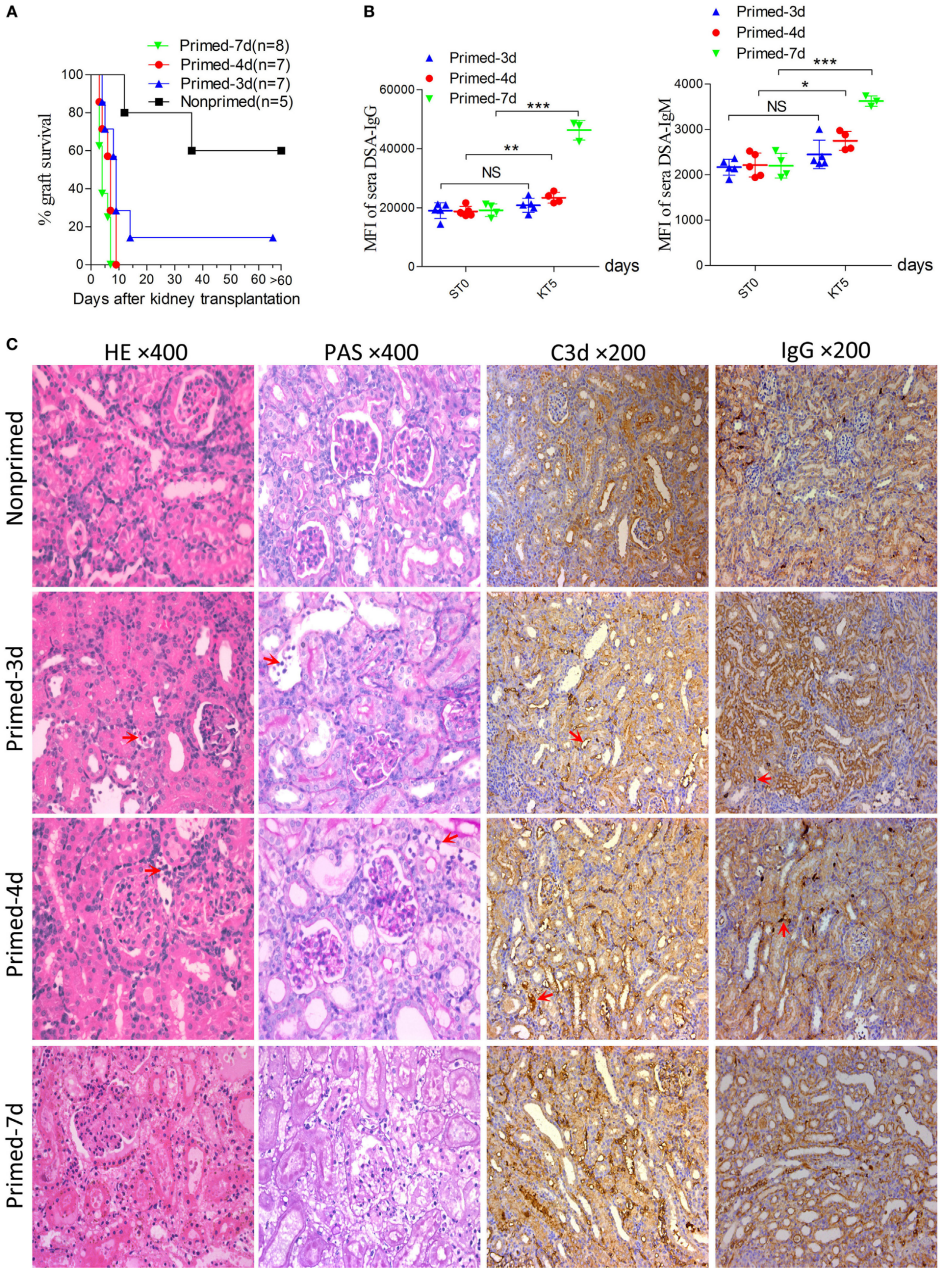
Modulating the robustness of renal allograft antibody-mediated rejection (AMR) by tuning the priming time at an early stage after skin sensitization. Balb/c recipient mice were primed with C3H skin grafts for 3, 4, and 7 days prior to receiving C3H donor kidneys. Balb/c mice receiving C3H kidneys without skin grafting acted as the nonprimed control. Donor-specific antibody (DSA) levels of recipient sera collected at time points ST0 and KT5 were measured by flow cytometry and expressed as mean fluorescence intensity (MFI). ST0 = day 0 before skin transplantation and KT5 = day 5 after kidney transplantation. **(A)** Kidney graft mean ± SD survival times were >31.2 ± 9.6, >9 ± 3.6, 6.4 ± 2.1, and 4.6 ± 1.6 days in nonprimed, primed-3d, primed-4d, and primed-7d groups, respectively. **(B)** DSA levels on KT5 decreased as the priming time reduced. Data represent the mean ± SD of at least three independent samples. *P < 0.05, **P < 0.01, ***P < 0.001 (Student’s *t*-test). NS, no significant difference. **(C)** Sections of kidney grafts on KT5 were stained with hematoxylin and eosin (H&E) and periodic acid-Schiff (PAS), ×400; and for IgG and C3d deposition, ×200. AMR histological features of tubular injury, peritubular capillary (PTC) dilation, and capillaritis, deposition of IgG and C3d in PTC were alleviated by decreasing the priming time. Arrows in HE and PAS staining indicate the PTC and capillaritis. Arrows in IgG and C3d staining indicate the positive depositions in PTCs.

An early kidney transplant within the 7-day time frame after skin priming had not been investigated and was investigated as an approach to reduce the AMR robustness of the subsequent kidney allograft. We therefore carried out kidney transplants at different times of donor skin priming to observe whether priming time can serve as a rheostat to regulate the severity of graft injuries caused by AMR within the 7-day time frame after skin priming. Recipient Balb/c mice were primed with C3H skin grafts prior to receiving C3H kidneys. Primed recipients were assigned to three groups based on the priming times of 3, 4, and 7 days, namely primed-3d, primed-4d, and primed-7d groups, respectively. Kidney transplants from C3H to nonprimed Balb/c mice served as the control group. The graft survival times were prolonged with decreased mean priming times, which were 4.6 ± 1.6, 6.4 ± 2.1, >9 ± 3.6, and >31.2 ± 9.6 days in Primed-7d, Primed-4d, Primed-3d, and Nonprimed groups, respectively. All renal grafts were rejected within 9 days after transplantation in the primed-4d and primed-7d groups, whereas two out of seven kidney grafts survived beyond 2 weeks and one of them survived over 60 days in the primed-3d group (Figure 2A).

Circulating DSA detection in recipient sera indicated that priming for 3 days with donor-matched skin grafts prior to kidney transplantation did not lead to markedly elevated DSA levels on day KT5 (KT# = day# after kidney transplantation) compared to the baseline levels on day 0 before skin transplantation (ST0). By contrast, recipient mice in both the primed-4d and primed-7d groups displayed significantly augmented DSA levels on day KT5 compared to the skin pre-transplant baseline level on day ST0 (Figure 2B). Kidney graft histology evaluated on day KT5 revealed that features typically associated with renal allograft AMR including acute tissue injury, peritubular capillary (PTC) dilation, microvascular inflammation, and deposition of complement split product C3d in PTCs were more salient in recipients receiving longer priming, whereas relatively mild to severe interstitial inflammation appeared as the prime renal histological manifestation in the nonprimed group and the typical cell-mediated rejection (CMR) histological feature of tubulitis was absent or mild (*t* score 0–1) in all groups (Figure 2C; Table 3).

**Table 3:** Summary of morphological findings in renal allografts.

**Groups**	**Animal#**	**pod**	**Histology**							**Overall histological interpretation**
			***i* score**	***t* score**	***v* score**	***g* score**	**ptc score**	**c3d staining**	**IgG staining**	
C3H-Balb/c, nonprimed	1	5	3	0	0	0	0	0	Minimal	IgG staining without evidence of rejection
	2	5	3	0	0	0	0	1	Minimal	IgG staining without evidence of rejection
	3	5	1	0	0	0	0	0	Negative	Nonspecific changes
	4	5	3	0	0	0	0	0	Negative	Nonspecific changes

C3H-Balb/c, primed-3d	5	3	0	0	0	0	0	0	Negative	Nonspecific changes
	6	3	0	0	0	0	1	0	Minimal	IgG staining without evidence of rejection
	7	5	1	0	0	0	2	2	Minimal	Active AMR
	8	8	3	1	0	0	0	1	Focal	CMR (borderline changes) mixed with active AMR
	9	13	2	1	0	1	1	3	Focal	Active AMR mixed with CMR (borderline changes)

C3H-Balb/c, primed-4d	10	3	0	1	0	0	2	2	Minimal	Active AMR mixed with CMR (borderline changes)
	11	5	1	0	0	1	2	2	Minimal	Active AMR
	12	5	1	0	0	1	3	3	Minimal	Active AMR
	13	7	1	1	0	1	3	1	Focal	Active AMR mixed with CMR (borderline changes)
	14	8	0	0	0	1	3	2	Focal	Active AMR

C3H-Balb/c, primed-7d	15	3	0	0	0	2	1	2	Minimal	Active AMR
	16	6	1	0	0	2	2	3	Focal	Active AMR
	17	6	1	0	0	1	1	2	Minimal	Active AMR
	18	5	0	1	0	3	1	3	Diffuse	Diffuse ATN
	19	5	1	1	0	2	3	3	Diffuse	Active AMR
	20	5	1	1	1	3	2	3	Diffuse	Diffuse ATN

C57BL/6-Balb/c, primed	21	5	2	0	0	1	0	1	Minimal	Active AMR
	22	15	2	0	0	3	0	2	Focal	Active AMR

C57BL/6-Balb/c, skin Tx	23	21	1	1	0	1	0	1	Minimal	CMR (borderline change) mixed with active AMR
	24	35	3	2	0	1	0	2	Focal	CMR IA mixed with active AMR
	25	35	3	2	0	0	1	2	Focal	CMR IA mixed with active AMR

*Histological changes were evaluated based on the Banff 2015 lesion grading system and classification categories: 0, absent; 1, mild; 2, moderate; 3, prominent*.

*pod, days after kidney transplantation; Tx, transplantation; ATN, acute tubular necrosis; AMR, antibody-mediated rejection; CMR, cell-mediated rejection*.

### Acute AMR Established by Kidney Transplantation at an Early Stage After Skin Priming Was Concomitant With a High Ratio of Graft-Infiltrating Macrophages

**Figure 3:**
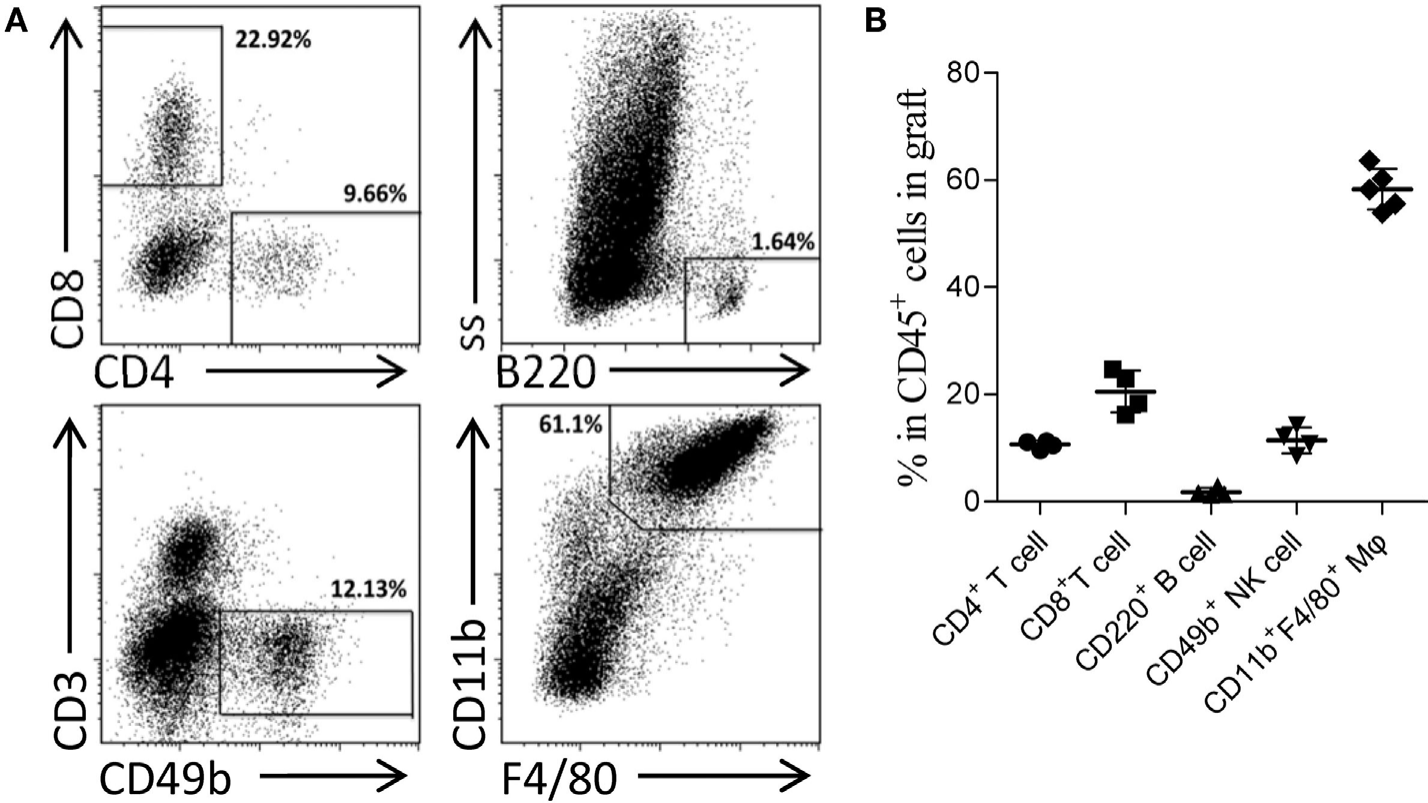
Acute antibody-mediated rejection (AMR) established by performing kidney transplant at early stage after skin priming was concomitant with a high ratio of graft-infiltrating macrophages. Balb/c recipient mice were primed with C3H donor skin grafts for 4 days prior to receiving the C3H kidney transplant to establish the AMR model. On day 5 after kidney transplantation, the phenotypes of CD45^+^ immune cells infiltrated in the kidney graft were determined by flow cytometry. **(A)** Representative dot plots of CD11b^+^F4/80^+^ macrophages, CD8^+^ T cells, CD49b^+^ NK cells, CD4^+^ T cells, and CD220^+^ B cells. **(B)** Macrophages form the most prominent population in the kidney graft, followed by CD8^+^ T cells, NK cells, CD4^+^ T cells, and B cell subsets. The results represent the mean ± SD of at least four independent samples.

Skin sensitization was not restricted to humoral response and also induced cellular alloimmunity. In this study, we utilized the primed-4d group to observe the cell infiltrations in AMR established by kidney transplantation at an early stage after skin priming. As shown in Figure 2C, although the typical cellular rejection histological change of tubulitis was absent or mild, interstitial inflammatory cell infiltrations (Table 3, *i* score 0–3) were observed in the renal graft on day KT5. Flow cytometry revealed that macrophages accounted for 58.3 ± 3.8% of the CD45^+^ immune cells infiltrated in the renal graft, followed by CD8^+^ T cells (20.5 ± 3.9%), NK cells (11.4 ± 2.4%), CD4^+^ T cells (10.7 ± 0.7%), and B cells (1.8 ± 0.7%) (Figure 3). These results indicate that macrophages were the predominant graft-infiltrating cells in this model on day KT5 after kidney transplantation.

### Improving Survival by Establishing the AMR Model in C57BL/6 to Balb/c Mice

**Figure 4:**
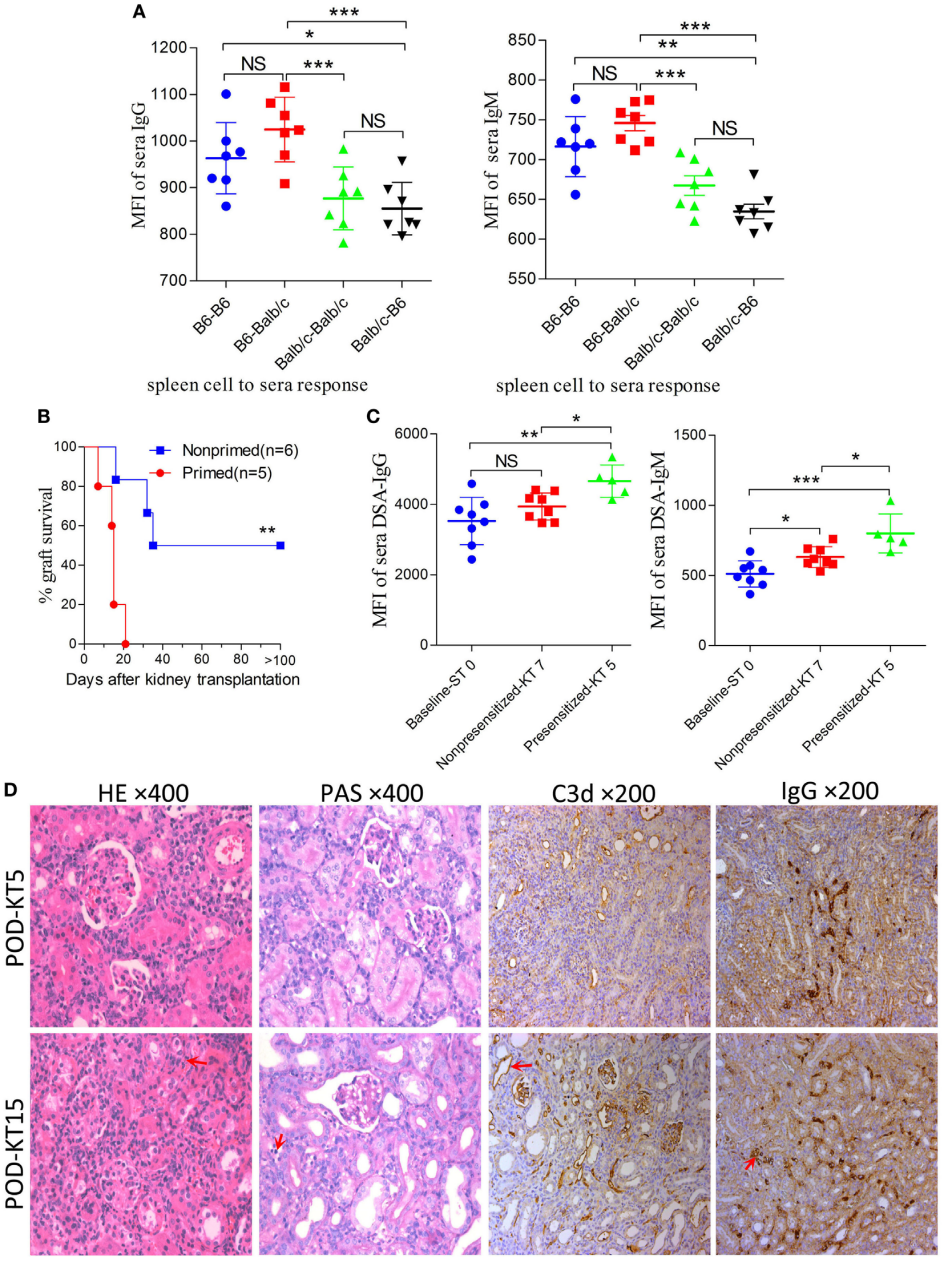
Improving survival by establishing an antibody-mediated rejection (AMR) model in C57BL/6 to Balb/c mice. Balb/c recipient mice were primed with C57BL/6 skin grafts for 4 days prior to receiving C57BL/6 kidney grafts. Balb/c mice receiving C57BL/6 kidneys without skin grafting acted as the nonprimed controls. Serum donor-specific antibody (DSA) levels were detected by flow cytometry and expressed as mean fluorescence intensity (MFI). The DSA data represent mean ± SD of at least five independent samples (Student’s *t*-test). ST0 = day 0 before skin transplant, KT5, KT7, and KT15 = day 5, 7, and 15 after kidney transplant, respectively. *P < 0.05, **P < 0.01, ***P < 0.001. NS, no significant difference. **(A)** Donor-reactive antibody baseline levels in C57BL/6-Balb/c were higher than those in Balb/c-C57BL/6. **(B)** Kidney graft mean ± SD survival times were >33.8 ± 9.3 and 14.4 ± 5.0 days in the nonprimed and primed groups, respectively (Log-rank test). **(C)** DSA levels of IgG and IgM in the sera from recipients in the primed group were significantly increased on KT5 compared to the baseline ST0, and were higher than the levels on KT7 in the nonprimed group. **(D)** Sections of kidney grafts were stained with hematoxylin and eosin (H&E) and periodic acid-Schiff (PAS), ×400; and for the deposition of IgG and C3d, ×200. AMR histological features of tubular injury, peritubular capillary (PTC) dilation, and capillaritis, deposition of IgG and C3d in PTC could be identified on KT5 and KT15 in the skin-primed group. Arrows in the HE and PAS staining indicate the PTC and capillaritis. Arrows in the IgG and C3d staining indicate the positive depositions in PTCs.

In the combination of C57BL/6 and Balb/c mice, the baseline cross allo-reactions of spleen cells to sera before skin graft priming were assessed and compared, and the unspecific binding of the iso-reaction of spleen cells to sera acted as the control. As showed in Figure 4A, differences in IgG or IgM baseline levels resulted from a discrepancy between donor spleen cells other than recipient sera from C57BL/6 or Balb/c mice. However, both IgG and IgM baseline levels of C57BL/6 to Balb/c allo-reaction were naturally higher than levels of Balb/c to C57BL/6. C57BL/6 and Balb/c mice were therefore adopted as donors and recipients, respectively.

Subsequently, the scheme of primed-4d by skin grafting, similar to that described above in the C3H to Balb/c combination, was applied to the C57BL/6 to Balb/c combination to set up a primed group; C57BL/6 to Balb/c kidney transplantation without skin graft priming was carried out to form a nonprimed control group. Our results revealed that renal grafts in the nonprimed group survived significantly longer than those in the primed group, with mean survival times >33.8 ± 9.3 vs. 14.4 ± 5.0 days (Figure 4B, *P* = 0.004). The survival time in primed recipients was significantly prolonged compared to that in the former C3H to Balb/c combination (14.4 ± 5.0 vs. 6.4 ± 2.1 days, *P* = 0.02). The outcome of DSA levels after kidney transplantation suggested that both the IgG and IgM levels in recipient sera from the primed group were significantly increased on day KT5 vs. ST0, and were higher than the levels on day KT7 in the nonprimed group (Figure 4C). Kidney graft histology evaluated on day KT5 and KT15 displayed the aforementioned described AMR features in the skin-primed group (Figure 4D; Table 3).

### Skin Priming After Kidney Transplantation Does Not Strongly Promote the Generation of DSAs

**Figure 5:**
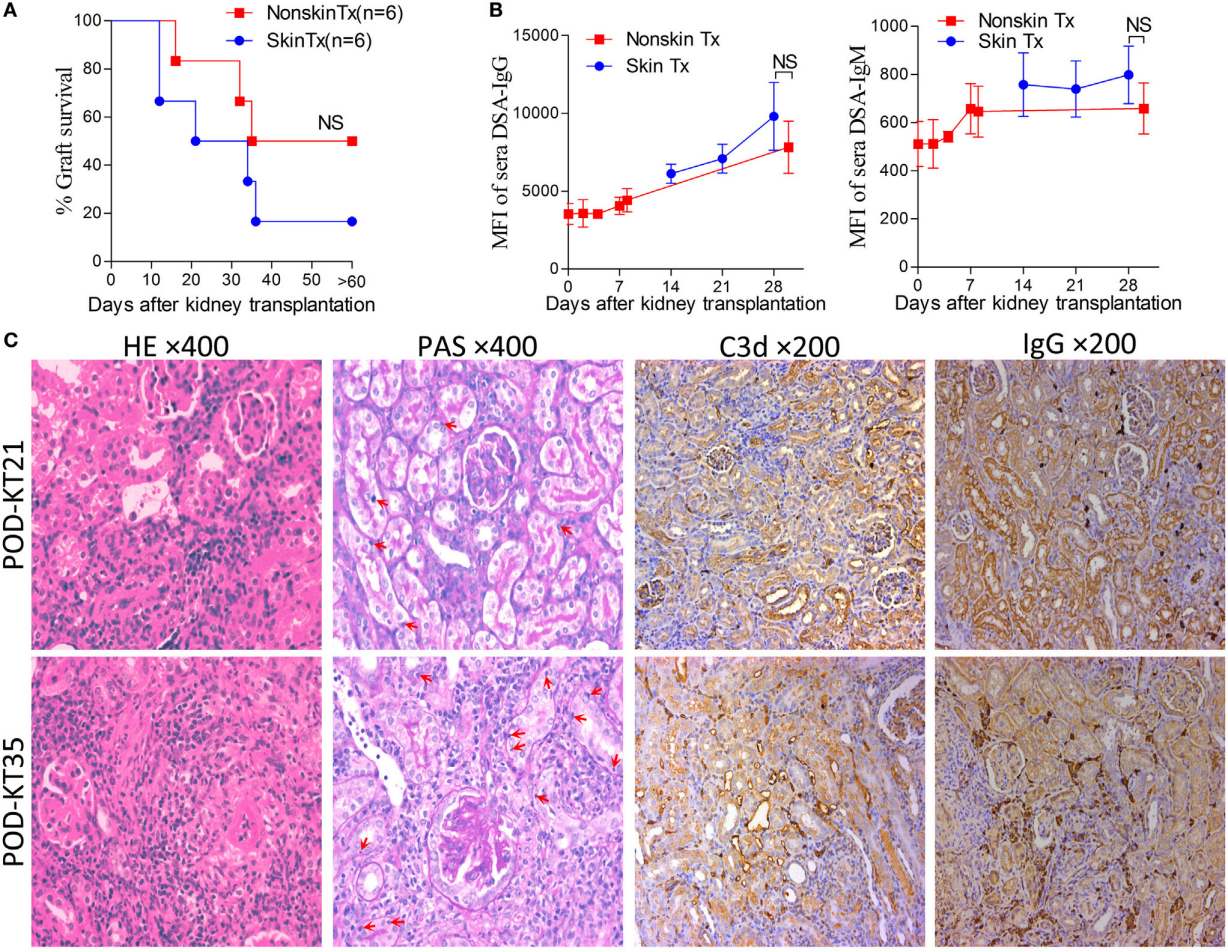
Skin priming after kidney transplant does not promote donor-specific antibody (DSA) generation strongly. C57BL/6 donor skin grafts were transplanted into Balb/c recipient mice on day 7 after the Balb/c mice received kidney grafts from C57BL/6 mice (skin Tx group). Balb/c mice that received C57BL/6 donor kidneys without subsequent skin grafting acted as controls (nonskin Tx group). **(A)** Donor-matched skin grafts after kidney transplantation decreased the renal graft survival time but not strongly within 60 days. **(B)** Recipient serum DSA levels were detected by flow cytometry and expressed as mean fluorescence intensity (MFI). The DSA data present the mean ± SD of at least six independent samples. Donor-matched skin grafts after kidney transplantation accelerated DSA generation but not strongly within 30 days. **(C)** Sections of kidney grafts after transplantation were stained with hematoxylin and eosin (H&E) and periodic acid-Schiff (PAS), ×400; and for the deposition of IgG and C3d, ×200. Histological features of interstitial inflammation and tubulitis (arrows) in cell-mediated rejection and deposition of IgG and C3d in peritubular capillaries could be identified on day 21 (KT21) and 35 (KT35) post kidney transplantation in the skin Tx group. NS, no significant difference.

In the clinical scenario, DSAs are also developed without sensitization before transplantation and frequently emerge gradually rather than acutely. There are compelling evidences that this type of chronic AMR is causally related to the destruction of clinical renal grafts (15–18). Given the repeated challenge by skin graft-induced DSA generation in monkeys which failed to raise DSAs after the first skin priming (19), it was speculated that skin priming after kidney transplantation may accelerate DSAs generation in recipient mice and the process may present as relatively moderate and chronic. To test this hypothesis, we transplanted C57BL/6 skin grafts to Balb/c recipient mice on day 7 after the Balb/c mice received C57BL/6 kidney grafts. Balb/c recipient mice transplanted with C57BL/6 kidneys without subsequently receiving skin grafts served as the controls. As shown in Figure 5, donor-matched skin grafts after kidney transplantation showed decreased renal graft survival times but not strongly within 60 days (Figure 5A, *P* = 0.24). Dynamic monitoring of DSA levels over 30 days after kidney transplantation demonstrated that challenge with donor-matched skin grafts after kidney transplantation slightly strengthened both DSA-IgG and IgM development but failed to reach significance (Figure 5B). Kidney graft histology evaluated on day KT21 and 35 showed primary cellular rejection (*i*1–3, *t*1–3) in the skin Tx group (Figure 5C; Table 3).

## Discussion

In this study, we provide evidence that the robustness of mouse renal allograft AMR induced by priming recipient to donor-specific skin grafts prior to kidney transplantation can be tuned by adjusting the priming time and altering the donor-recipient combination at an early stage after skin priming. First, we found that DSAs induced by skin sensitization were affected by the donor-recipient combination and skin graft size, and were sustained for a long time in mice. Next, we established a model with less severe renal allograft AMR based on a reported pair of C3H and Balb/c mice; we then demonstrated that the most common laboratory mice, C57BL/6 and Balb/c, can also be used to successfully generate a model of renal allograft AMR. Finally, we demonstrated that a donor-matched skin challenge after renal transplantation did not strongly accelerate DSA development.

In the skin transplant models used in the present study, as shown in Figure 1, use of skin grafts of 8–10 × 10 mm^2^ from the donor tail as the stimulus resulted in similar patterns of circulating DSA generation in the recipients from the four different donor-recipient combinations in the early 7 days after skin priming, with a rather flat trend of DSA-IgG levels. This feature was in line with other studies on mice and rats (9, 20). In these studies, the investigators reported that the DSA-IgG level did not begin to rise until day 5 after priming the recipient with the donor abdominal skin graft from DAB/2 to C57BL/6 mice (9), and failed to change until day 3 after skin priming from Brown Norway to Lewis rats (20). These results were observed because the activation process of B cells to DSA-secreting plasma cells takes time, given that the B cell responses are processed through a series of conversions, resulting in the generation of memory B cells or antibody-secreting plasma cells with the added features of somatic mutation and class-switch recombination (21, 22). After 7 days, the peak time and the fold increase at peak time to the baseline of the DSA-IgG level was varied in different combinations, suggesting that the donor-recipient combination determined the DSA development pattern after skin priming. The results on DSA-IgM generation after priming the recipient with skin graft of 8–10 × 10 mm^2^ (Figure 1) revealed a pattern similar to that described by other investigators (10), which exhibited a fluctuation without significantly ascending with time.

Skin from the ear and back has more Langerhans cells compared to that from the tail and provokes a more robust immune response (23). The size of non-vascularized skin grafts was found to be inversely correlated with donor chimerism (24). Thus, it was believed that the skin priming effects also varied with the priming recipient to the different sizes or origins of the skin grafts. In this study, skin grafts of 4–5 × 5 mm^2^ triggered a more striking priming effect than those of 8–10 × 10 mm^2^ at an early stage (Figure 1B). This suggested that 4–5 × 5 mm^2^ skin grafts from the donor tail were capable of providing sufficient antigens for B cell activation, and that the later rise start point of DSA-IgG levels in recipient primed with 8–10 × 10 mm^2^ skin grafts may result from a greater consumption of DSA-IgG in reaction to a larger skin graft *per se*.

The patterns of DSA generation after skin priming in this study supported those observed by previous investigators performing kidney transplantation at 1–3 weeks after priming the recipient with skin grafts (9–11, 20), or at even longer priming times (11, 12, 19) to establishing preexisting DSA renal allograft AMR models. However, these models tended to develop highly severe graft histological changes and alteration in priming times ranging from 7 to 91 days in these models failed to alter the severity of renal graft injuries in mice, which may make the AMR irreversible and compromise the use of this model to study AMR intervention strategies (9–12). Our results in the C3H to Balb/c combination showed similar results: recipients primed for 14 days survived indifferently from those primed for 7 days before renal transplantation (data not shown). Although the mechanism underlying this remains largely unclear, the outcomes of this study provide preliminarily insights. As shown in Figure 1, after the DSA-IgG level achieved a peak, it did not return to the pre-priming level within 100 days after priming, indicating that the preexisting alloantibodies were sufficient to destroy the kidney grafts equally at any time after the 7-day priming within 100 days.

However, the outcomes of kidney grafts transplanted within 7 days of the early stage after skin priming are still unknown, which may decrease the severity of renal graft injuries due to a shorter skin priming time and undetectable circulating DSA before renal transplantation (Figure 1). By using the 8–10 × 10 mm^2^ of donor tail skin grafts, in the early 7-day stage after skin priming, we examined whether the outcome of subsequent kidney transplantation can be modulated by adjusting the priming time. The kidney allograft survival improved when the skin priming time prior to kidney transplantation was decreased (Figure 2A). DSA detection in the sera and histological changes in the kidney grafts based on Banff classification (14) revealed that the improved kidney graft survival resulted from an attenuated graft injury mediated by DSAs (Figures 2B,C; Table 3). In addition, some recipients in the primed-3d group survived comparably to those in the nonprimed group (Figure 2A), suggesting that B cells may fail to mount acute AMR after kidney transplantation if the priming time is scant. In this context, all renal grafts were rejected within 9 days after transplantation in the primed-4d and primed-7d groups, and renal allograft histological results from the primed-7d groups at KT5 were too severe to model AMR under a clinical setting (Figure 2C). The model established using the primed-4d protocol was thus believed to be an optimized renal allograft AMR model in this study.

Intriguingly, in this optimized model, circulating DSAs were only detectable post kidney transplantation, which is quite different from previous reports on AMR in mice (6, 7, 9–12). Current strategies such as plasmapheresis or immunoabsorption to eliminate the existing circulating DSAs in the clinic are hard to implement in mice. This model provides an excellent tool to explore novel strategies directed against B cell activation or antibody-secreting plasma cells after kidney transplantation, but avoid consideration for eliminating preexisting DSAs. It has also been used in our recent studies on exploring AMR intervention strategies (25, 26).

In clinical practice, based on our study and investigations from others, acute AMR is commonly concomitant with acute cellular rejection (27, 28). The currently reported renal allograft AMR in mice are also mixed AMR models (9–11). In this study, due to the short interval between skin sensitization and kidney transplantation and the fact that skin grafts, as strong stimuli for allo-sensitization, induce both cellular and humoral alloimmunity in the optimized primed-4d group, involvement of cellular responses is also observed by the inflammatory infiltration of multiple immune cells, especially macrophages, in the graft (Figure 3). These findings were consistent with the renal allograft AMR in rats (29, 30). Increased macrophage infiltration in renal allograft AMR is also observed in mice (9, 10) and in the clinical scenario (31, 32). The underlying causes of macrophage infiltration are still not well understood. Our recent study (26) showed that kidney graft-infiltrating macrophages during AMR predominantly adopted the M1 phenotype rather than the M2 phenotype. Considering that IFN-γ produced by Th1 cells drives macrophages to activate the M1 phenotype (33) and that Th1 cell infiltration in grafts was predominant during kidney AMR (34), it is possible that Th1 cell infiltration in the grafts is a key reason for macrophage infiltration during AMR. The pro-inflammatory factors produced by M1 macrophages are detrimental to graft survival. However, it is also described in mice renal allograft AMR that T cells are the most frequently infiltrating cells in renal grafts, followed by macrophages/monocytes, and B cells (9). Accordingly, our model also exhibited high ratio of graft-infiltrating macrophages with AMR playing a dominant role in recipient demise. The typical CMR histological change of tubulitis based on Banff criteria (14) was absent to borderline in all skin graft primed groups, whereas the AMR histological features of peritubulitis and the deposition of C3d and IgG in PTCs were prominent (Figure 2; Table 3).

Although mouse renal allograft AMR models had been reported by several study groups (6, 7, 9–11), they have not been investigated in the most commonly used strain combination of C57BL/6 and Balb/c mice (4, 35). It is worthy to establish an AMR model using these strains given their relatively low cost and high availability in most laboratories, which may tremendously facilitate studies associated with renal allograft AMR. We tested the feasibility of using this combination to construct an AMR model. Studies harnessing this model may benefit from its prolonged recipient survival time, which can offer a longer time for intervention in AMR. Together with the results from skin transplant alone in this study, our data confirmed that strain combination is another key factor for the mouse renal allograft AMR model.

In mice that accepted the kidney allografts, secondary challenge with donor splenocytes failed to stimulate a hypersensitivity response (36). Despite the robust immunogenicity of skin grafts relative to others (37), it also failed to strongly promote greater generation of DSAs by priming recipients with donor-matched skin grafts after kidney transplantation in this study. Simultaneous kidney-skin transplantation also failed to significantly affect kidney graft survival (data not shown). However, repeatedly challenging the recipient with donor-specific skin does trigger DSA generation in non-human primates if the first skin priming fails to stimulate DSA production in the recipient (19). The mechanism underlying this phenomenon is not clear, considering that the likely tolerogenesis of kidney grafts coexists with its allogeneity.

In summary, in this study, we showed that early renal transplantation on day 4 after skin graft priming is a feasible approach to establish a mouse renal allograft AMR model with reduced robustness of AMR. C57BL/6 and Balb/c mice can also be used for setting up such a disease model. We also presented the long-term DSA changes after skin graft sensitization for the first time and explored the DSA changes when the skin-kidney graft sequence was reversed. The outcomes from the present study provide insights for understanding mouse renal allograft AMR models and may facilitate future AMR associated studies.

## Ethics Statement

This study was carried out in accordance with the recommendations of the animal use protocol, which was approved by the Institutional Animal Care and Use Committee of Sun Yat-sen University (Approve Number: 160520).

## Author Contributions

QS and DZ designed the research; DZ, TL, SL, YZ, HZ, JZ, FH, and YD performed the experiments; DZ, TL, YZ, JZ, and YD analyzed the data; DZ, TL, and SL wrote the manuscript. All the authors read and approved the final manuscript.

## Conflict of Interest Statement

The authors declare that the research was conducted in the absence of any commercial or financial relationships that could be construed as a potential conflict of interest.

## Abbreviations

AMR, antibody-mediated rejection; ATN, acute tubular necrosis; CMR, cell-mediated rejection; DSA, donor-specific antibody; DAB, diaminobenzidine; H&E, hematoxylin and eosin; MFI, mean fluorescence intensity; PAS, periodic acid-Schiff; PBS, phosphate buffered saline; PTC, peritubular capillary.
